# Structure-based Discovery of Novel Small Molecule Wnt Signaling Inhibitors by Targeting the Cysteine-rich Domain of Frizzled*

**DOI:** 10.1074/jbc.M115.673202

**Published:** 2015-10-26

**Authors:** Ho-Jin Lee, Ju Bao, Ami Miller, Chi Zhang, Jibo Wu, Yiressy C. Baday, Cristina Guibao, Lin Li, Dianqing Wu, Jie J. Zheng

**Affiliations:** From the ‡ Department of Structural Biology, St. Jude Children's Research Hospital, Memphis, Tennessee 38105,; the § Department of Ophthalmology, Stein Eye Institute, David Geffen School of Medicine, University of California, Los Angeles, Los Angeles, California 90095,; the ¶ State Key Laboratory of Molecular Biology, Institute of Biochemistry and Cell Biology, Shanghai Institutes for Biological Sciences, Chinese Academy of Sciences, Shanghai 200031, China, and; the ‖ Department of Pharmacology, Yale University School of Medicine, New Haven, Connecticut 06510

**Keywords:** anticancer drug, drug discovery, molecular docking, nuclear magnetic resonance (NMR), Wnt signaling

## Abstract

Frizzled is the earliest discovered glycosylated Wnt protein receptor and is critical for the initiation of Wnt signaling. Antagonizing Frizzled is effective in inhibiting the growth of multiple tumor types. The extracellular N terminus of Frizzled contains a conserved cysteine-rich domain that directly interacts with Wnt ligands. Structure-based virtual screening and cell-based assays were used to identify five small molecules that can inhibit canonical Wnt signaling and have low IC_50_ values in the micromolar range. NMR experiments confirmed that these compounds specifically bind to the Wnt binding site on the Frizzled8 cysteine-rich domain with submicromolar dissociation constants. Our study confirms the feasibility of targeting the Frizzled cysteine-rich domain as an effective way of regulating canonical Wnt signaling. These small molecules can be further optimized into more potent therapeutic agents for regulating abnormal Wnt signaling by targeting Frizzled.

## Introduction

Wnt signaling controls cell fate, proliferation, migration, tissue architecture, and organogenesis during embryonic development (1–7). Canonical Wnt signaling is initiated by the binding of secreted Wnt proteins with the membrane co-receptors LRP5/6 (low density lipoprotein receptor-related protein 5/6) and FZD (Frizzled). Subsequent Dishevelled activation dissembles the adenomatous polyposis coli/axin/glycogen synthase kinase 3β complex, which results in β-catenin accumulation and the formation of β-catenin/transcription factor complexes in the nucleus, leading to the transcription of multiple downstream genes (8). Dysregulation of the canonical Wnt signaling pathway has been observed in many diseases including bone diseases, diabetes, and several types of cancer (9).

**FIGURE 1.**
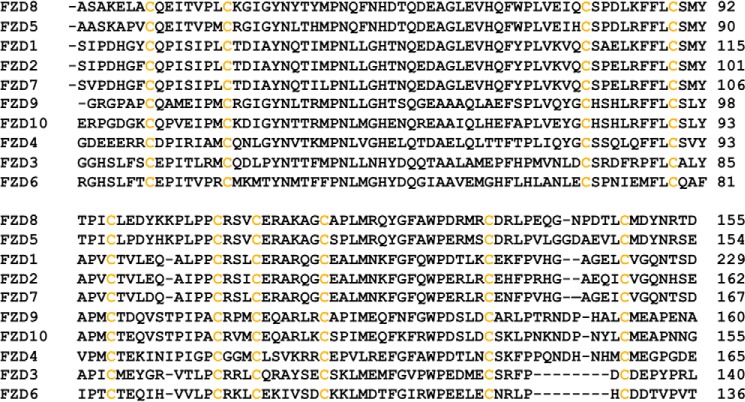
**Primary sequence alignment of mouse FZD CRDs.** The sequence alignment was generated using ClustalW2.

FZDs are G protein-coupled receptor-like proteins. They consist of an extracellular N terminus containing a highly conserved cysteine-rich domain (CRD; Fig. 1),^4^ a seven-transmembrane domain, and a cytoplasmic C terminus (10, 11). To date, 10 human FZDs, FZD1–FZD10, have been cloned and characterized (10, 11). Wnt glycoproteins initiate Wnt signaling by interacting with the FZD CRD, as well as with the extracellular domains of LRP5/6 to form a Wnt/FZD/LRP complex. sFRPYs (secreted Frizzled-related proteins) inhibit Wnt signaling by blocking the interaction between Wnt proteins and FZD with a homologous CRD domain, demonstrating that FZD is a critical target for upstream Wnt signaling regulation (12, 13). Moreover, because overexpression of FZD has been observed in many cancers (14–19), the CRD has been proposed as a potential target for therapeutic development against cancer and other human diseases. Indeed, it was shown that a monoclonal antibody that targets the CRD domains of FZDs can block canonical Wnt signaling induced by Wnt ligands and is able to reduce the growth of different types of tumor (9).

Here, we report on the discovery of small molecule inhibitors of the FZD8 CRD using a hybrid structure-based lead discovery approach that combined molecular modeling, biophysical methods, and a cell-based assay. The crystal structure of the CRD of mouse FZD8 has been reported, and mutagenesis studies identified the key residues for the FZD CRD binding to Wnt ligands (20). A recent x-ray structure of *Xenopus* Wnt8 (XWnt8) in complex with the mouse FZD8 CRD confirmed that this site directly interacts with the XWnt8 ligand and plays a key role in mediating Wnt signaling (20, 21). Based on the structural information, several hierarchical virtual screening (VS) simulations were conducted to select small molecule compounds that can target the Wnt binding site on the FZD8 CRD. We tested 120 top ranked compounds for their inhibitory activities of canonical Wnt signaling by using a cell-based luciferase reporter assay. Five compounds were shown to be strong inhibitors of canonical Wnt-β-catenin signaling. In addition, using NMR spectroscopy, we confirmed that these small molecule compounds specifically bind, with low micromolar affinities, to the Wnt/FZD binding site on the FZD8 CRD. Our work demonstrates that interrupting the Wnt-FZD CRD interaction by small molecules from structure based drug discovery is of highly promising potential for the development of new therapeutics against diseases influenced by abnormal Wnt signaling.

## Experimental Procedures

### 

#### 

##### Docking and Scoring in Virtual Screening

The Glide module in the Schrödinger package was used for the virtual screening of the CRD of mouse FZD8 (Frizzled8). Two small molecule databases, National Cancer Institute plate 2007 (containing 117,500 compounds) and ChemDiv (containing 1,414,174 compounds), were used for structure-based virtual screening. The ChemDiv database was prescreened using the UNITY module (Tripos) to identify compounds that are potentially compatible with the size and shape of the primary Wnt binding site on the mouse FZD8 CRD (21). This procedure reduced the database size of ChemDiv to 11,000 compounds. All selected compounds were prepared using the LIGPREP module in the Schrödinger package (Schrödinger, New York, NY). The pH was set at 7.5, and OPLS 2005 force field parameters were applied to all selected small molecules. The receptor model was derived from the mouse FZD8 CRD crystal structure (Protein Data Bank code 1IJY, chain A) (20) with removal of all water molecules. The protein preparation script in MAESTRO (Schrödinger, New York, NY) was used to add polar hydrogen atoms, partial charges and fix errors in crystal structure. The molecular mechanics force field grids around the space of primary Wnt binding site were generated by Glide, and the virtual screening was performed by Glide with standard precision. The compounds with top predicted binding free energies and reasonable binding orientations within the defined active site were subsequently obtained from NCI and ChemDiv for further evaluation of the docking results (22).

##### Cell Culture

A stable transfected cell line expressing Luciferase under a transcription factor (TCF)/LEF promoter was used. 3T3 cells were maintained in 5% CO_2_ at 37 °C in Dulbecco's modified Eagle's medium containing 10% fetal bovine serum, 4.5 g/liter d-glucose, 2 mm glutamine, 0.1 mm nonessential amino acids, 10 mm HEPES, 100 U/ml penicillin, and 100 μg/ml streptomycin. Cells were seeded and grown overnight in a 96-well plate at 4 × 10^5^ cells/ml confluences. Wnt3a and compounds (0–40 μm) were dissolved in DMEM (Invitrogen) assay media containing 0.5% FBS. The plate was divided between two sections: one with 50 ng/ml of Wnt3a and a control with the assay media. Cells were incubated with protein plus compound between 12 and 16 h before readings. Each experiment was performed in duplicate for each compound.

##### Luciferase Assays

The Promega (Madison, WI) ONE-Glo^TM^ + Tox luciferase reporter and cell viability assay kit was used to measure inhibition of Wnt signaling. In brief, cells were first incubated for 30 min with a cell-permeant fluorogenic substrate. In a living cell, the substrate is cleaved by a liver protease that results in emission of fluorescence. This was used to normalize for potential toxic effects of the ligands. After measuring cell viability, 5′-fluoroluciferin was added to the cells. In the cells, the fluoroluciferin is cleaved by luciferase, which emits luminescence. Both fluorescence and luminescence were measured using an EnVision® microplate reader. Analysis of inhibitors dose-response data and calculation of IC_50_ values were performed using the program Origin.

##### Western Blotting Analysis

Human embryonic kidney cell line (HEK293T) cells were seeded in 24-well plate at a density of 10 × 10^4^ cells/well and cultured for 24 h. Cells were stimulated by Wnt3a conditioned medium supplemented with 10 μm individual compounds or DMSO (control) for 3 h. Cells were lysed in Nonidet P-40 buffer (50 mm Tris-HCl, 150 mm NaCl, 5 mm EDTA, and 1% Nonidet P-40). Lysates were cleared by centrifugation, and protein concentrations were determined by BCA assay. Protein samples were dissolved on 6% SDS-PAGE gel and then transferred to 0.45 μm nitrocellular membranes. Membranes were blocked by 5% nonfat milk and then incubated with anti-phosphor-LRP6 (S1490) antibody (Cell Signaling Technology, Inc.; catalog no. 2568s) (1:2000) overnight at 4 °C, followed by anti-rabbit HRP secondary antibody (1:2000) for 1 h at room temperature. The membranes were washed with TBST for three times at room temperature. Finally, phosphor-LRP6 band was detected with the enhanced chemiluminescence substrate (Thermo Fisher Scientific Inc.). Tubulin was used as the loading control. ECL signal was calculated by using National Institutes of Health ImageJ software.

##### Expression and Purification of the Mouse FZD8 CRD

The mouse FZD8 CRD (Ala^28^–Asp^155^) was subcloned into pET28a vector and was transformed into Rosetta2 (DE3) cells (Novagen). The cells were grown in MOPS media supplemented with [^15^N]ammonium chloride and [^13^C]glucose as the source of nitrogen and carbon, respectively. The cells were grown to a *A*_600_ value of 0.5 ∼ 0.6 and induced with 1 mm isopropyl β-d-1-thiogalactopyranoside for 16 h at 37 °C with 200 rpm. The target protein was expressed insolubly as inclusion bodies. To denature the target protein, the pellet of 1 liter of *Escherichia coli* cell culture was dissolved with 20 ml of 8 m urea with 100 mm β-mercaptoethanol and then was refolded by rapid dilution using 10 mm Tris, pH 9.0, with 50 mm Arg and 1 mm oxidized glutathione. The protein was further purified by HPLC and was dialyzed in 50 mm potassium phosphate (pH 6.5 or 7.5) with 5 mm EDTA. The two-dimensional ^1^H-^15^N HSQC spectrum of the purified ^15^N-labeled FZD8 CRD showed that the FZD8 CRD protein is folded (23).

##### Biolayer Interferometer (BLI) Experiments

The Octet RED instrument (FortéBio) was used to measure the interaction of mouse FZD8 CRD to the compounds identified from the both the VS and the cell-based assays. Super streptavidin (SSA) sensors were used to attach the biotinylated FZD8 CRD. Biotinylation of FZD8 CRD was made by incubating 100 μl of 2 mm EZ-Link Sulfo-NHS-LC-Biotin (Thermo Scientific, catalog no. 21335) with 1 ml of 8 μm FZD8 CRD in 50 mm potassium phosphate buffer, pH 6.5, for 4 h on a rocking platform at room temperature. A desalting column was used to purify the biotylated-FZD8 CRD. Before experiments, the SSA sensors were preincubated for 10 min in 50 mm potassium phosphate buffer, pH 6.5, with 0.01% Tween 20, 5% DMSO, and 1 mg/ml BSA (assay buffer). The biotinylated proteins were attached to the SSA sensors for 10 min. The excess protein was removed by washing with assay buffer for 20 min. To determine the binding affinity of mouse FZD8 CRD to the five compounds, several concentrations of compounds were used. The association step for 50 s was followed by the dissociation step for 100 s. Biocytin-loaded SSA sensor without loading biotinylated protein was used as a control to correct the systematic optical artifacts and baseline drifts (24). All steps were performed at 25 °C with 1,000 rpm rotary shaking. The processed data were fitted locally with the integrated fitting function using the 1:1 binding model in Forté Bio analysis software (v6.4). The kinetic constants *k*_on_ (on rate constant), *k*_off_ (off rate constant), and *K_D_* were calculated from curve fitting.

##### Preparation of Compound Solutions for NMR Studies

The compounds were dissolved in DMSO-*d*_6_ to make 10 mm stock solutions. To investigate physical properties, such as solubility and self-aggregation, of compounds in aqueous buffer, we made a series of samples with various compound concentrations in 50 mm potassium phosphate at two different pHs, pH 6.5 and 7.5, with 0.5 mm EDTA, 10% D_2_O, and 5% DMSO-*d*_6_. Because of the poor solubility of compounds, we were unable to make the desired concentration of 12.5–200 μm. We obtained one-dimensional ^1^H NMR spectra of each compound at various desired concentrations. The peak intensity changes of resonance for each compound were examined to determine the solubility, as well as visual inspection of precipitation of compound in buffer. The solubility of compounds in the two different buffers was examined.

##### NMR Experiments

All spectra were recorded using either ^1^H,^15^N-labeled or ^1^H-, ^15^N-, and ^13^C-labeled protein on Bruker Avance 600- or 800-MHz NMR spectrometers equipped with ^1^H/^15^N/^13^C detect, triple resonance cryogenic inverse probes at 25 or 32 °C. All spectra were processed using Topspin 3.0 NMR software (Bruker Biospin) and analyzed using the program CARA (computer-aided resonance assignment). To assign the backbone chemical shift of mouse FZD8 CRD, 200 μm FZD8 CRD was prepared in 50 mm potassium phosphate, pH 6.5, and 10% D_2_O (v/v). We performed two-dimensional ^1^H-^15^N HQSC and three-dimensional HNCA, HNCOCA, CACB (CO)NH, HNCACB, and ^15^N NOESY (mix time = 120 ms) NMR experiments at 32 °C. Chemical shift perturbation (CSP) experiments were performed using ^15^N-labeled mouse FZD8 CRD. The two-dimensional ^1^H-^15^N HSQC (or ^1^H-^15^N fast HSQC) (25) spectra were recorded as a function of concentration of compound. A stock solution of 10 mm compound was made in DMSO-*d*_6_ for use in NMR titration experiments. The concentration of DMSO in the NMR titration experiment was below 3%. A control experiment was done by titrating 5% DMSO, which did not show any structural change in this condition. The chemical shift perturbation (Δδ, ppm) of ^1^H and ^15^N resonances were obtained and weighted according to Equation 1.

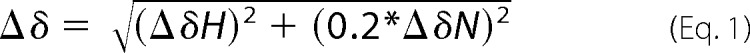


## Results

### 

#### 

##### Hierarchical Structure-based VS for FZD 8 CRD Inhibitor Discovery

**FIGURE 2.**
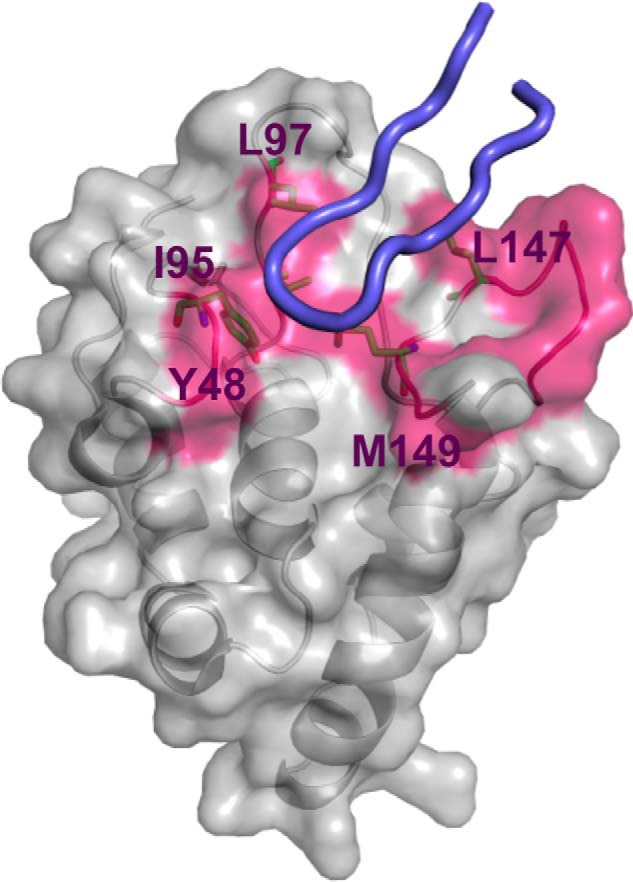
**Residues and surface on the FZD8 CRD defined for virtual screening.** The residues critical for Wnt signaling activity are shown in *magenta*, whereas the residues defined for UNITY searches are shown as sticks (model derived from coordinates of Protein Data Bank code 4F0A). The bound conformation of index finger loop region (amino acids 308–336) of XWnt8, which is critical for Wnt-FZD CRD recognition (21), is shown in *blue cartoon* for the validation of potential cavity determination.

The atomic structure of the FZD8 CRD was reported previously, and the residues critical for mediating Wnt signaling were examined by mutagenesis experiments (20). It was shown that *Xenopus* Wnt8 (XWnt8) binds to a cavity formed by residues Ile^46^, Gly^47^, Tyr^48^, Ile^95^, Leu^97^, Gln^141^, Gly^142^, Asn^143^, Pro^144^, Asp^145^, Thr^146^, Leu^147^, Met^149^, Asp^150^, and Tyr^151^ of the mouse FZD8 CRD, which is located at the FZD8 CRD dimerization interface in the crystal structure (Protein Data Bank code 1IJY). A recent x-ray structure of the Wnt-FZD CRD complex confirmed that this cavity directly binds to a loop structure known as the “index finger” region on the Wnt8 protein (21) (Fig. 2). Based on the available structural information, hierarchical structure-based VS utilizing the UNITY module in the SYBYL package (Tripos, Inc.) and Glide (Schrödinger) was performed. A chemical diversity (ChemDiv) library (containing ∼1,414,000 compounds) and a NCI 2007 plate library (containing ∼117,000 compounds) were searched for potential small molecule compounds that would bind to the FZD8 CRD Wnt binding site (26).

The initial predocking screening was performed by UNITY (Tripos, Inc.). A UNITY three-dimensional query was built by defining the hydrophobic region formed by residues Tyr^48^, Ile^95^, Leu^97^, Leu^14^7, and Met^149^ (Fig. 1), all of which are critical for Wnt-FZD8 CRD binding. After searching both libraries using UNITY, ∼5,000 compounds were selected to be docked into the Wnt binding cavity using Glide. By visual inspection of bound conformations and comparing predicted binding free energies, we selected the top 40 candidate compounds from the ChemDiv library and the top 80 compounds from the NCI 2007 plate library as the initial virtual hits. These virtual hits had the best predicted binding free energies with reasonable bound conformations.

##### Five Compounds Show Dose-dependent Inhibition of Wnt3a-induced Signaling in a Cell Assay

**FIGURE 3.**
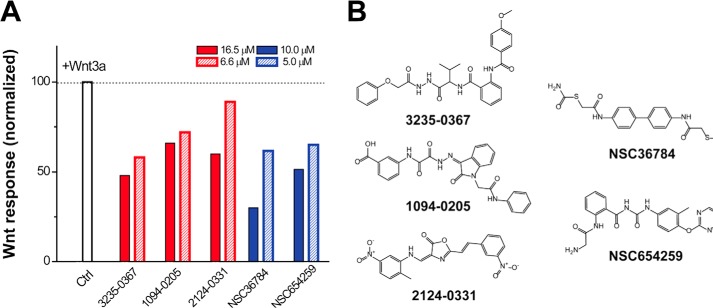
**Inhibition of Wnt signaling by small molecule compounds.** *A*, 3T3 cells were treated with 50 ng/mol recombinant Wnt3a. The level of the Wnt-β-catenin pathway response according to luciferase activity was measured in the absence (*white bar*) or presence of a compound (*red bars*, ChemDiv compounds; *blue bars*, NCI compounds) at two different concentrations. The Wnt activity was normalized against the cells treated with empty vehicle (same amount of DMSO). Five compounds inhibit the Wnt3a-induced β-catenin pathway in a concentration-dependent manner. *Ctrl*, control. *B*, chemical structure of the compounds that inhibited Wnt signaling in the cell-based assay.

Because the Wnt-FZD CRD interaction mediates canonical Wnt signaling from upstream of the pathway by directly binding to Wnt proteins, a compound competing with Wnt proteins by binding the FZD CRD with high affinity will reduce the Wnt-β-catenin signaling activity. Wnt3a is the best studied canonical Wnt ligand (27). To test potential inhibitory effects of compounds selected from VS on Wnt-β-catenin signaling, we performed a luciferase-based screening assay on Wnt-3a mediated reporter gene activity in 3T3 cells (28). Initially, each compound was incubated at two different concentrations in 3T3 cells transfected with a Wnt3a-conditioned medium that activates the Wnt pathway. Of the 120 compounds tested, five compounds exhibited strong inhibition of Wnt3a-induced responses in a concentration-dependent manner (Fig. 3).

**FIGURE 4.**
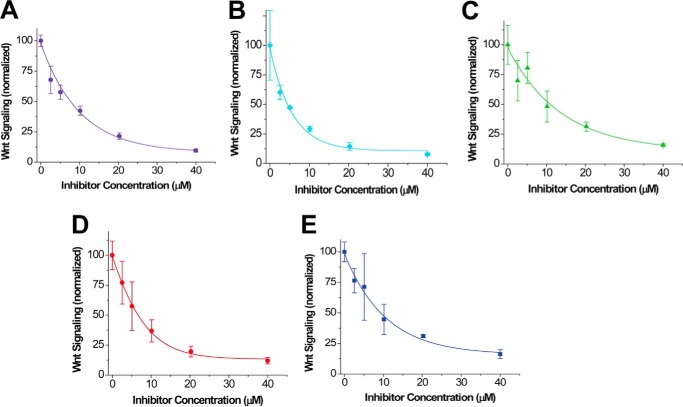
**Dose-dependent Inhibition of Wnt3a-induced β-catenin signaling by the identified compounds.** 3T3 cells were treated with Wnt3a. *A–E*, dose response curves of the inhibitory effect on Wnt/β-catenin signaling with five compounds: 3235-0367 (*A*), 1094-0205 (*B*), 2124-0331 (*C*), NSC36784 (*D*), and NSC654259 (*E*). The Wnt activity was normalized against the cells treated with empty vehicle (same amount of DMSO). The IC_50_ value of each compound is summarized in Table 1.

We then explored the dose dependent inhibitory effect of each compound identified from initial screening in the same cell-based assay with compound concentration gradients up to 40 μm and accordingly calculated the IC_50_ (half-maximal inhibitory concentration) values. These results are summarized in Table 1. The results show that all five compounds have the capability to inhibit Wnt3a-induced β-signaling at the low micromolar range dose-dependently (Fig. 4) with compounds NSC36784 (Fig. 4*D*) and NSC654259 (Fig. 4*E*) being the most potent inhibitors.

**TABLE 1:** **The IC_50_ value and binding affinity (*K_D_*) of compounds to mouse FZD8 CRD by cell-based assays and BLI assays, respectively**

Compounds	IC_50_*^a^*	*K_D_**^b^*
	μ*m*	μ*m*
3235–0367	7.1 ± 1.4	2.5 ± 0.4
1094–0205	5.0 ± 1.1	3.4 ± 1.4
2124–0331	10.4 ± 2.0	2.3 ± 1.0
NSC36784	6.5 ± 0.9	3.3 ± 2.9
NSC654259	5.7 ± 1.2	2.9 ± 2.4

*^a^* Cell-based assays. Duplicate experiments were performed.

*^b^* 50 mm potassium phosphate, pH 6.5, with 0.01% Tween 20, 1 mg/ml BSA.

##### All Five Compounds Inhibit LRP6 Phosphorylation Induced by Wnt Signaling

**FIGURE 5.**
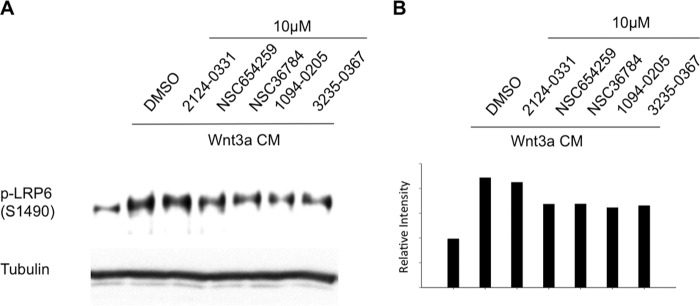
**Inhibition of Wnt induced LRP6 phosphorylation by the five identified compounds.** *A*, HEK293T cells were seeded in a 24-well plate for 24 h and were then treated with Wnt3A CM along or with 10 μm of the five compounds, as well as DMSO as control, respectively, as indicated for 3 h. The lysates of the cells were immunoblotted for pLRP6 Ser^1490^ and tubulin. *B*, densitometric qualification of pLRP6 Ser^1490^ normalized to tubulin.

A critical event in canonical Wnt signaling is phosphorylation of the Wnt co-receptors, LRP5/6. Upon stimulation with Wnt, the intracellular domain LRP6 is phosphorylated at multiple sites including Thr^1479^, Ser^1490^, and Thr^1493^ (29–32). We therefore used the LRP6 phosphorylation as a marker to further assess the ability of the compounds to inhibit Wnt signaling. In HEK293T cells, increased phosphorylation level at Ser^1490^ of LRP6 could be clearly observed after Wnt3A treatment. When we added the compounds to the cell cultures, respectively, we found that comparing to the empty carrier, DMSO, all five compounds reduced phosphorylation of LRP6 at Ser^1490^ (Fig. 5) confirming that, indeed, all five compounds are inhibitors of the canonical Wnt signaling pathway.

##### All Five Compounds Bind to the FZD CRD at Micromolar Range

**FIGURE 6.**
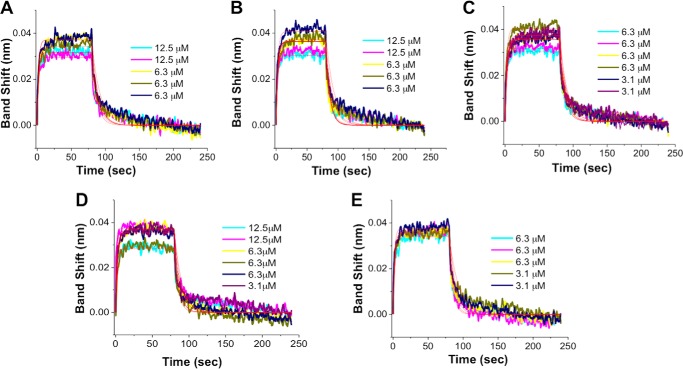
**BLI binding assays show the binding of five compounds to FZD8 CRD.** The SSA sensors with biotinylated mFZD8 CRD were exposed to several different concentrations (3.1–12.5 μm) in 50 mm potassium phosphate, pH 6.5, with 5% DMSO-*d*_6_, 0.01% Tween 20, and 1 mg/ml BSA (assay buffer). The solubility of compound was visually inspected once diluted in the assay buffer. *A*, 3235-0367. *B*, 1094-0205. *C*, 2124-0331. *D*, NSC36784. *E*, NSC654259. The processed data were fitted locally with the integrated fitting function by a 1:1 binding mode (*red line*). The respective *K_D_* values obtained by curve fitting were summarized in Table 1.

To further evaluate the five compounds, we generated mouse FZD8 CRD and measured the dissociation constants (*K_D_*) of selected compounds to the FZD8 CRD by BLI experiments. To avoid the low solubility issue of some of the compounds, relatively low concentrations of the compounds were used in the experiments. For each compound binding to the biotinylated FZD8 CRD, the binding assays were repeated multiple times (all the experiments are reported in Fig. 6), and the average *K_D_* value of each was obtained (Table 1). All five compounds bound to the FZD8 CRD show *K_D_* values at low micromolar values, which is consistent with the outcomes of cell-based assay and NMR experiments.

##### All Five Inhibitors Bind to the Primary Wnt Binding Site on the FZD8 CRD

**FIGURE 7.**
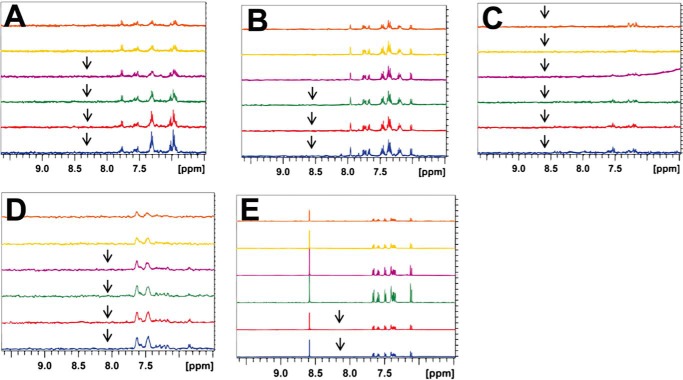
**Solubility test of compounds in phosphate buffer.** *A–E*, ^1^H NMR spectra of compounds with different desired concentration in 50 mm potassium phosphate, pH 6.5. *Orange*, 12.5 μm; *yellow*, 25 μm; *purple*, 50 μm; *green*, 100 μm; *red*, 150 μm; *blue*, 200 μm. A stock solution of 10 mm compound was prepared in DMSO-*d*_6_. The *arrows* are placed to indicate that precipitations of compound were observed during the NMR experiment at the concentration. *A*, 3235-0367. *B*, 1094-0205. C, 2124-0331. *D*, NSC36784. *E*, NSC654259.

**FIGURE 8.**
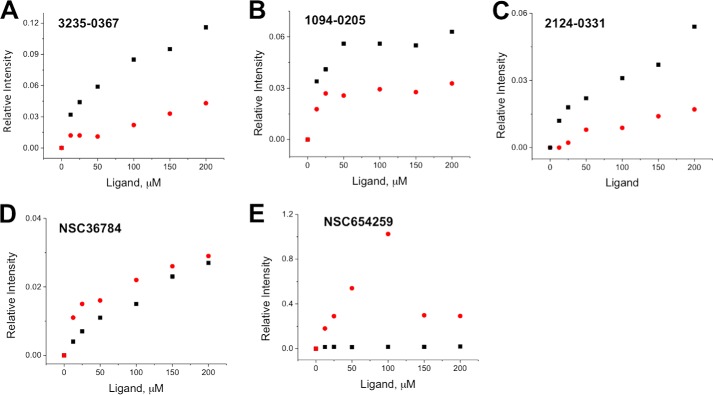
**Solubility test of compounds in phosphate buffer.** Relative intensity change of compounds for ^1^H NMR spectra as a function of concentration in 50 mm potassium phosphate, pH 6.5 (*red dots*) or pH 7.5 (*black squares*) with 5% DMSO-*d*_6_ and 10% D_2_O. From the results, we were able to estimate the solubility of each compound. *A*, 3235-0367; *B*, 1094-0205; *C*, 2124-0331; *D*, NSC36784; *E*, NSC654259.

We then used NMR spectroscopy to study the interactions between the five compounds and the FZD8 CRD. Because the typical required sample concentration for NMR is relatively high, the issue of compound solubility was addressed before NMR studies were performed. In the study, the compounds were dissolved in DMSO-*d*_6_ to make 10 mm stock solutions. We made a series of samples with various compound concentrations in 50 mm potassium phosphate at two different pHs, pH 6.5 and 7.5, with 0.5 mm EDTA, 10% D_2_O, and 5% DMSO-*d*_6_. We then obtained one-dimensional ^1^H NMR spectra of each compound at desired concentrations of 12.5–200 μm. The peak intensity changes of resonance for each compound were examined to determine the solubility, as well as visual inspection of precipitation of compound in buffer (Figs. 7 and 8). The solubility of compounds in the two different buffers was examined. Overall, all five compounds are more soluble at pH 6.5. Therefore, all the NMR experiments were performed at pH 6.5.

**FIGURE 9.**
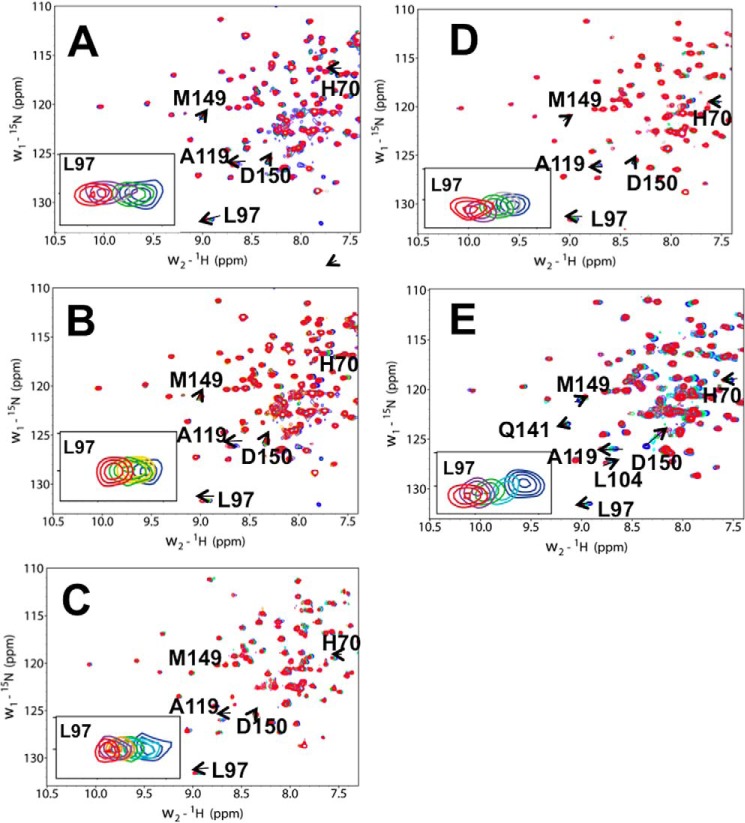
**NMR analysis of compound binding to the mouse FZD8 CRD.** Shown are the extended ^1^H-^15^N HSQC spectra of FZD8 CRD in the absence (*blue*) and the presence of compound (*red*). Compounds 3235-0367 (*A*), 1094-0205 (*B*), 2124-0331 (*C*), NSC36784 (*D*), and NSC654259 (*E*) were titrated into the solution of ^15^N-labeled mouse FZD8 CRD, respectively. The *insets* show the extended two-dimensional ^1^H-^15^N HSQC spectra of residue Leu^97^ at different concentrations of compound.

**FIGURE 10.**
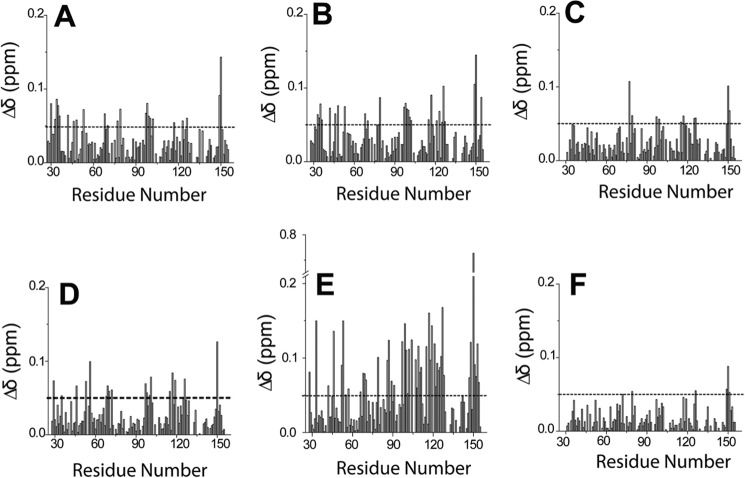
**The CSPs of the FZD8 CRD induced by different inhibitors.** *A*, 3235-0367. *B*, 1094-0205. *C*, 2124-0331. *D*, NSC36784. *E*, NSC654259; *F*, 2% (v/v) DMSO.

We then performed chemical shift perturbation experiments to identify the residues of FZD8 CRD involved in binding (33). The two-dimensional ^1^H-^15^N HSQC spectra of ^15^N-labeled FZD8 CRD were recorded during the titration of compounds into a solution of the CRD. All five compounds induced CSPs in the ^1^H-^15^N HSQC spectra of the FZD8 CRD (Fig. 9). Among the five compounds, NSC654259 showed the largest overall CSPs in ^1^H-^15^N HSQC spectra (Figs. 9 and 10). The signal of residues Leu^97^, Met^149^, and Asp^150^ of the FZD8 CRD are among those residues that have chemical shift changes induced by the binding during the titration of all compounds into the solution of ^15^N-labeled FZD8 CRD (Fig. 9). As expected, these residues correspond to the primary protein-protein binding site of the FZD8 CRD. The signal of residue Leu^97^ disappeared and reappeared upon stepwise addition of compound 3235-0367, indicating that the complex formation is in the intermediate exchange range on the NMR time scale and the binding affinity is in the submicromolar range.

##### Structural Details of the Identified Compounds Binding to the FZD8 CRD

**FIGURE 11.**
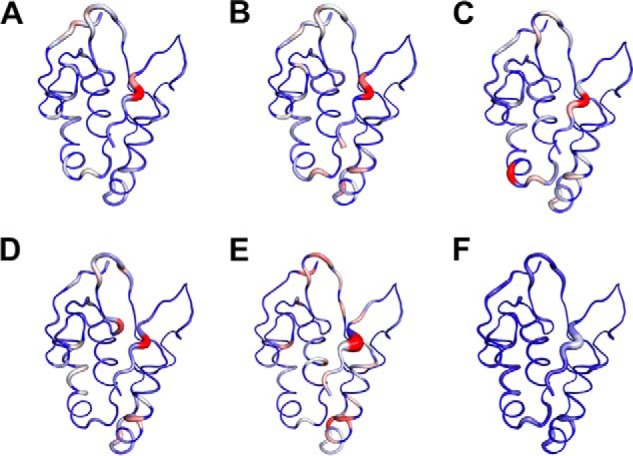
**Mapping the binding site of compounds on the FZD8 CRD.** Compounds that bind to the primary protein-protein interaction site of FZD8 CRD. *A–E*, ribbon representation of the backbone structure of mouse FZD8 CRD in complex with each compound. *A*, 3235-0367. *B*, 1094-0205. *C*, 2124-0331. *D*, NSC36784. *E*, NSC654259. *F*, 2% DMSO. The backbone thickness of the ribbon diagram is directly proportional to the weighted sum (in ppm) of the ^1^H and ^15^N chemical shifts on binding to each compound. The program PyMOL was used to generate all figures.

**FIGURE 12.**
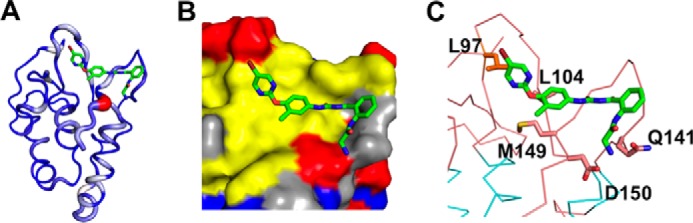
**Docking structure of FZD8 CRD with NSC654259.** *A*, ribbon structure of FZD8 CRD in complex with NSC654259. *B*, binding surface of FZD8 CRD in complex with NSC654259. The hydrophobic residues are shown in *yellow*; positive residues are in *blue*; and negative charge residues are in *red. C*, key residues of FZD8 CRD bound to NSC654259 (*green*, carbon; *red*, oxygen; *blue*, nitrogen) are shown in stick model with labeling. Hydrogen atoms are omitted for clarification.

To better understand of the binding mode of each compound, we analyzed the docking structures of identified compounds with the data obtained in the CSP experiments. We first generated a ribbon diagram of the backbone structure of the FZD8 CRD based on the CSP data (Fig. 11), wherein the thickness of ribbon is proportional to CSP values observed upon binding. The ribbon diagram of the FZD8 CRD clearly shows that all five compounds bind to the primary protein-protein binding site of the FZD8 CRD. Moreover, we observed an additional salt bridge forming between the side chain of Asp^150^ with the primary amine group of NSC654259 in the docking model (Fig. 12). This predicted interaction is consistent with the observation of extra CSP of Asp^150^ in two-dimensional HSQC spectra (Fig. 9) when titrating NSC654259 with the FZD8 CRD. Interestingly, each compound induces the different chemical shift perturbations at the residues Leu^97^, Lys^102^, and Asp^150^ at the FZD8 CRD. This implies that each compound may be in different orientations when binding to the FZD8 CRD (Fig. 10). For two compounds, 1094-0205 and 2124-0331, we also observed some CSPs at the secondary lipid-binding site of the FZD8 CRD (21) (Fig. 9, *B* and *C*). These changes may be the result of induced conformational change upon binding of compounds. Overall the predicted docking structures of the identified compounds are very consistent with two-dimensional HSQC CSP data.

## Discussion

Several components of the Wnt signaling pathway have been extensively targeted for the development of new therapeutics to treat diseases such as cancer (6, 34–37). Lithium, which is a GSK3β inhibitor, effectively decreases cell proliferation and induces nonapoptotic cell death in Wnt subtype medulloblastoma, in which Wnt signaling is up-regulated by accumulation of intracellular β-catenin (38). Nonsteroidal anti-inflammatory drugs are also capable of inhibiting Wnt/β-catenin pathway (39). Small molecules regulating canonical Wnt signaling by targeting the Dishevelled PDZ domain (22, 39, 40) and the LRP5/6 extracellular domains (26) have also been identified.

In this study, we aim to develop Wnt inhibitors by targeting FZD. FZD is the first discovered Wnt protein receptor and is crucial for initiation of Wnt signaling (12). FZD dysregulation is an important biomarker in many tumor types including lung and colorectal cancers and hepatocellular carcinoma (18, 41–43). An antibody (OMP-18R5) interacting with FZD extracellular domain directly blocks canonical Wnt signaling and inhibits the growth and tumorigenicity of multiple human tumors (9). Here we describe a systematic structure-based small molecule lead discovery for targeting the FZD8 CRD by the combination of hierarchical VS, cell-based assays, BLI, and NMR spectroscopy. From hierarchical VS and cell-based assays, we identified five compounds capable of inhibiting the Wnt3a-induced β-catenin signaling in the low micromolar range of IC_50_ values. We further demonstrated that these compounds antagonize canonical Wnt signaling by binding to the primary Wnt binding site of the FZD8 CRD with submicromolar binding affinities from BLI experiments. To our knowledge, the compounds reported here are the first reported small molecule canonical Wnt signaling inhibitors that specifically target the Wnt-FZD CRD interaction.

There are 19 Wnt ligands and 10 FZD receptors in mouse and human, and the combination of the Wnt-FZD interactions can be complex and functionally redundant, which poses a challenge for the development of effective inhibitors (9). Not surprisingly, the sequence alignment of mouse FZD CRDs indicates that residues in the primary protein-protein binding site of FZD, which may determine the specificity of the Wnt-FZD CRD interaction, are diverse (Fig. 1) (21). However, the residues Leu^97^, Met^149^, and Asp^150^ in the FZD8 CRD, which are critical for binding, are conserved in many FZD CRDs (Fig. 1). This suggests that the compounds identified here may also bind to other FZD receptors, and it would be interesting to see whether the compounds can indeed target CRDs other than the FZD8 CRD (23, 44). On the other hand, the chemical diversity of the highly potent small molecule Wnt signaling antagonists identified also provides an excellent platform for specifically targeting individual FZD CRD by lead optimization. Therefore, our study could be the starting point for the development of an array of different small molecule inhibitors for different FZD CRD; and such compounds will be very useful in dissecting roles of different FZD receptors in different Wnt signaling pathways.

In the PubChem BioAssay database, it is reported that one of the compounds we identified, NSC654259, has antitumor activity in multiple types of tumor cell lines. Although we cannot rule out that such an antitumor effect is due to its target other than FDZ CRDs, given that the a monoclonal antibody that targets the FZD CRDs is able to reduce the growth of different types of tumor (9), the information does lead us to speculate that FZD CRD may be a druggable target of great therapeutic potential for treating diseases with aberrant Wnt signaling. The genetic alteration of Wnt signaling in cancer is very diverse, making it impractical to develop a single effective solution for all genetic variations (37). To explore potential benefits of combinatorial therapy, it is vital to gain access to compounds targeting multiple important Wnt signaling components. The discovery of novel FZD CRD inhibitors may add important candidates to the Wnt signaling regulator pool and will be valuable for further understanding Wnt signaling and discovering most effective therapeutic combinations by targeting abnormal Wnt signaling based on genetic profiles of individuals. Moreover, besides the cost factor associated with antibody drugs (45, 46), another advantage of FZD CRD inhibitors is that they may have a broader spectrum of the targets; perhaps a small molecule inhibitor drug can be developed to target most, if not all, of the 10 human FZDs.

## Author Contributions

J. Z. conceived and coordinated the study. H.-J. L., J. B., A. M., and J. Z. wrote the paper. J. B. and A. M. performed computational studies. H.-J. L. and C. G. generated proteins. H.-J. L., J. B., and A. M. performed biophysical studies. C. Z., J. W., Y. C. B., L. L., and D. W. performed cell studies. All authors reviewed the results and approved the final version of the manuscript.

## Footnotes

4 The abbreviations used are:

CRD: cysteine-rich domain
VS: virtual screening
CSP: chemical shift perturbation
BLI: biolayer interferometry
SSA: super streptavidin.
